# Investigating the induction of polyphenol biosynthesis in the cultured *Cycolocarya paliurus* cells and the stimulatory mechanism of co-induction with 5-aminolevulinic acid and salicylic acid

**DOI:** 10.3389/fbioe.2023.1150842

**Published:** 2023-03-09

**Authors:** Li-Juan Ling, Meng Wang, Chuan-Qing Pan, Dao-Bang Tang, En Yuan, Yuan-Yuan Zhang, Ji-Guang Chen, Da-Yong Peng, Zhong-Ping Yin

**Affiliations:** ^1^ Jiangxi Key Laboratory of Natural Products and Functional Foods, College of Food Science and Engineering, Jiangxi Agricultural University, Nanchang, China; ^2^ Sericultural & Agri-Food Research Institute, Guangdong Academy of Agricultural Sciences/Key Laboratory of Functional Foods, Ministry of Agriculture and Rural Affairs/Guangdong Key Laboratory of Agricultural Products Processing, Guangzhou, China; ^3^ College of Pharmacy, Jiangxi University of Traditional Chinese Medicine, Nanchang, China

**Keywords:** plant cell engineering, *Cyclocarya paliurus*, polyphenol, co-induction technology, metabolome, transcriptome

## Abstract

**Background:** Plant cell culture technology is a potential way to produce polyphenols, however, this way is still trapped in the dilemma of low content and yield. Elicitation is regarded as one of the most effective ways to improve the output of the secondary metabolites, and therefore has attracted extensive attention.

**Methods:** Five elicitors including 5-aminolevulinic acid (5-ALA), salicylic acid (SA), methyl jasmonate (MeJA), sodium nitroprusside (SNP) and *Rhizopus Oryzae* Elicitor (ROE) were used to improve the content and yield of polyphenols in the cultured *Cyclocarya paliurus (C. paliurus)* cells, and a co-induction technology of 5-ALA and SA was developed as a result. Meanwhile, the integrated analysis of transcriptome and metabolome was adopted to interpret the stimulation mechanism of co-induction with 5-ALA and SA.

**Results:** Under the co-induction of 50 μM 5-ALA and SA, the content and yield of total polyphenols of the cultured cells reached 8.0 mg/g and 147.12 mg/L, respectively. The yields of cyanidin-3-O-galactoside, procyanidin B1 and catechin reached 28.83, 4.33 and 2.88 times that of the control group, respectively. It was found that expressions of TFs such as *CpERF105, CpMYB10* and *CpWRKY28* increased significantly, while *CpMYB44* and *CpTGA2* decreased. These great changes might further make the expression of *CpF3′H* (flavonoid 3′-monooxygenase), *CpFLS* (flavonol synthase), *CpLAR* (leucoanthocyanidin reductase), *CpANS* (anthocyanidin synthase) and *Cp4CL* (4-coumarate coenzyme A ligase) increase while CpANR (anthocyanidin reductase) and *CpF3′5′H* (flavonoid 3′, 5′-hydroxylase) reduce, ultimately enhancing the polyphenols accumulation

**Conclusion:** The co-induction of 5-ALA and SA can significantly promote polyphenol biosynthesis in the cultured C. *paliurus* cells by regulating the expression of key transcription factors and structural genes associated with polyphenol synthesis, and thus has a promising application.

## 1 Introduction

Polyphenols are one of the most diverse and widely distributed secondary metabolites in the plants (Grajeda-Iglesias et al., 2022). Catechins, anthocyanins, flavonoids, and procyanidins are four common categories of polyphenols with a diversity of bio-activities such as anti-oxidation, anti-senescence, anti-tumor and anti-diabetic, and therefore have a promising application prospect in foods, cosmetics and pharmaceuticals (Cao et al., 2017; Khan and Siddiqui, 2017; Zong et al., 2020).

At present, polyphenols are mainly obtained from plant materials such as leaves, stems, flowers and fruits by extraction and purification (Cui et al., 2014). There are lots of plants that can be used in plant cell culture to produce polyphenols, but plants have a long growth cycle and a vulnerability to the natural environment and pests (Liu et al., 2022). It is also costly to get polyphenols of high purity because of a considerable variety of components in the differentiated plant organs like leaves and flowers (Silva et al., 2015), which restricts the availability of natural plant polyphenols (Dea et al., 2017). Over the past century, plant cell and tissue culture technologies have developed rapidly and shown outstanding advantages in the plant secondary metabolites production, such as short production cycle, independence of natural conditions and conveniences in extraction and purification (Davies et al., 2013; Regine et al., 2018). The output of shikonin derivatives reached 2,300 mg/L by cell suspension cultures of Lithospermum erythrorhizon (Yasuhiro and Yasuhiro, 1985). Amacha cells were cultivated in a 15L jar fermentor to bio-synthesize polyphenols such as skimmin and hydrangenol-8-O-β-D-glucose (Suzuki et al., 2014). These reports indicate that using plant cell culture to produce valuable secondary metabolites has promising application.

Although there are many reports about secondary metabolites production by plant cell culture, only a few of them have been produced in large-scale, such as paclitaxel (Li et al., 2009), ginseng (Hu et al., 2001) and shikonin (Sormeh et al., 2016) *etc.* Other species, however, cannot be utilized on a large scale probably due to the low contents and yields of active compounds. For instance, the yields of catechin, caffeic acid, kaempferol and apigenin in the cultured date palm cells were only 155.9, 162.7, 89.7, and 242.7 μg/L, respectively (Naik and Al-Khayri, 2018). Kikowska et al. (2019) found the contents of polyphenols in the shoot cultures of *Eryngium alpinum* L like caftaric acid, caffeic acid, and chlorogenic acid were only 48.6, 27.1 and 139 μg/g, respectively. Consequently, it is urgent to improve the target compound yields of the cultured plant cells.

In order to promote polyphenol production by plant cell culture, researchers have been searching for effective methods to improve the target polyphenol output, such as optimization of culture conditions including light (Curtin et al., 2003), carbon source (Ochoa-Villarreal et al., 2015) and medium composition (Naik and Al-Khayri, 2018). Precursor feeding, for example, phenylalanine (Li et al., 2021), and elicitors like 5-ALA (Zheng et al., 2018) and 24-Epibrassinolide (Tassoni et al., 2012). Among the above-mentioned promotion strategies, elicitation is regarded as one of the most effective ways, and now has attracted extensive attention (Rs et al., 2020). According to the report of Jeandet et al. (2016), resveratrol yield reached 7 g/L in grape suspension cells under the co-stimulation of MeJA and acyclodextrin. Li et al. (2021) also found that under the co-induction of SA and Phe, the total polyphenol content and yield were as high as 41.36 mg/g and 752.93 mg/L, respectively.

*Cycolocarya paliurus* is a unique and endangered plant with a sporadic distribution in many provinces of southern China (Zhao et al., 2020). Researches have shown that the leaves of this plant contain a wealth of bio-active compounds such as tirterpenoids, polyphenols, and polysaccharides, and therefore display multiple physiological activities and pharmacological functions, including anti-diabetes (Lin et al., 2020), anti-hyperlipidemia (Yao et al., 2015), and anti-oxidation (Yao et al., 2015). Polyphenols are the important bio-active components in the leaves of *C. paliurus*, which endow it with various pharmacological activities (Xiong et al., 2016). However, polyphenols in *C. paliurus* have not aroused much interest and therefore not been fully utilized at present. This may be due to the great difficulties in seed germination and asexual propagation of this plant that limit the availability of *C. paliurus* resources.

*C. paliurus* cell culture is a potential way to bio-synthesize these bio-active polyphenols. Our laboratory has been engaged in the researches related to the cell and tissue culture of *C. paliurus* for more than 15 years, screened and cultivated six categories calluses with unique characteristics like crisp light yellow-green callus (CYG), compact green callus (CG), wet grayish brown callus (WGB), crisp white callus (CW), crisp light pink callus (CPC), and red callus (CR), among which CYG was the most common and typical line, while CR was extremely rare (Zhao et al., 2020). CR was found accidentally during our long-term continuous sub-culturing and screening of *C. paliurus* callus, and its growth, morphology and secondary metabolites were all significantly different from the most common CYG. Our previous studies showed that CR was rich in polyphenols, including anthocyanins, catechin, procyanidin B1 and its content were much higher than that in CYG and other four typical callus (Zhao et al., 2020). Relatively speaking, our *C. paliurus* callus also displayed a higher polyphenol content than that induced from some other reported plants (Naik and Al-Khayri, 2018), and therefore has the potential to produce polyphenols with high antioxidant activity. Consequently, a stable cell suspension culture was successfully developed using CR to bio-synthesize polyphenols.

In the present paper, five elicitors including 5-ALA, SA, MeJA, SNP, and ROE were used to increase the content and yield of polyphenols in the cultured *C. paliurus* cells, meanwhile the co-induction mechanism of 5-ALA and SA was further investigated based on the integrated analysis of transcriptome and metabolome. This paper provides a theoretical basis for the regulation of polyphenol production by *C. paliurus* cell suspension culture.

## 2 Materials and methods

### 2.1 Materials and chemicals

*C. paliurus* suspension cells were cultured and preserved in the Jiangxi Key Laboratory of Natural Products and Functional Food, Jiangxi Agricultural University (Nanchang, China). ROE was obtained from Guangdong Microbial Culture Collection Center. Murashige and Skoog (MS) medium (Murashige and Skoog, 1962) was bought from Hope Bio-Technology Co., Ltd. (Qingdao, China). Kinetin (KT), 1-naphthylacetic acid (NAA), 5-Aminolevulinic acid (5-ALA) and 2,4 -dichlorophenoxy acetic acid (2,4 -D) were offered by Aladdin Co., Ltd. (Shanghai, China). Other chemicals (purity: AR) were supplied by Sigma-Aldrich Co., Ltd. (Shanghai, China).

### 2.2 Cultivation of *C. paliurus* cells

*C. paliurus* calluses with excellent appearance and loose texture were inoculated into a 100 mL culture flask containing 40 mL MS medium supplemented with 1.0 mg/L kT, 0.3 mg/L NAA, 0.3 mg/L 2,4 -D, 30 g/L sucrose and 0.7% agar. The cultures were cultivated in a rotary orbital shaker at 115 rpm and (25 ± 2)°C under constant light, and sub-cultured with an inoculation amount of 6.0 g per culture bottle every 7 days.

### 2.3 Biomass determination of the cultured cells

The cultured cells were collected on the 6^th^ day using suction filtration, and then dried in an oven at 50°C until a constant weight was achieved. The weight of the harvested cells (dry weight, and DW) was measured after being cooled down to the room temperature, which was recorded as a biomass increment indicator (DW).

### 2.4 Polyphenol extraction and determination

#### 2.4.1 Polyphenol extraction and standard solution preparation

Polyphenol extraction was performed according to Zhao et al. (2020). 1 g dried cell powder was extracted by 30 mL ethanol supplemented with 1% hydrochloric acid (v/v) in an ultrasonic extractor at 50°C for 60 min. The extracts were centrifuged at 6,000 r/min for 10 min, and then the supernatant was collected and filtered through a 0.22 μm filtration membrane. The standards (procyanidin B1, (+)-catechin, cyanidin-3-*O*-galactoside) were dissolved in 1% (v/v) hydrochloric acid water to prepare 1.0 mg/mL of standard solution, which were used to prepare series of standard solutions with concentrations from 10 to 400 μg/mL, 20–400 μg/mL, 5–200 μg/mL, respectively.

#### 2.4.2 Polyphenol determination

Polyphenol extraction and standard solution were determined with HPLC (1260 HPLC system, Agilent Technologies, United States) on a C18 column (4.6 × 250 mm, 5 μm). The assay procedure was performed in accordance with that described by (Zhao et al., 2020). In this paper, the sum content and yield of the three main components (procyanidin B1, (+) - catechin, cyanidin-3-*O*-galactoside) was calculated and used as total polyphenol content (TPC) and total polyphenol yield (TPY) of the cultured *C. paliurus* cells. The standard curves for the detection of polyphenols were shown in Supplementary Table S1.

### 2.5 Preparation of elicitors

Referring to Li et al. (2021), MeJA and SA were dissolved in ethanol while SNP and 5-ALA were dissolved in distilled water, which were further filtrated through a 0.22 μm membrane and used as elicitors to stimulate the biosynthesis of polyphenols. ROE was prepared according to the method described by O-Hernández et al. (2017). The collected mycelium pellets were fully ground with phosphate buffer solution (PBS, pH = 6.0), and then the ground mixture was centrifuged at 4,000 r/min for 10 min. Afterward the supernatant was collected and sterilized at 121°C for 25 min, which was used as ROE in the following induction experiments. The concentration of ROE was characterized with total sugar content, which was determined by Anthrone-sulfuric method using glucose as standard (Dona et al., 2011).

### 2.6 Single-factor experiment for elicitor screening

In the evaluation of the promotion effects of each elicitor on the polyphenol biosynthesis in the cultured cells, the effects of concentration and addition time of the elicitors were all taken into consideration, which were designed and described in the Supplementary Table S2.

### 2.7 Optimization experiment of co-induction parameters

The co-induction concentrations of 5-ALA and SA were optimized by comprehensive test (completely random design), using TPC as the evaluation indicator. The test concentration of 5-ALA and SA were designed and described in Supplementary Table S2. The addition and harvest time of the elicitors were as mentioned in the Supplementary Table S2.

### 2.8 Metabolome detection and analysis

#### 2.8.1 Extraction of metabolites

The cultured fresh cells of the control group and treated group co-induced by 5-ALA and SA were sampled on the 6^th^ day, and these samples were frozen with liquid nitrogen quickly and then stored at −80°C for the subsequent detection. Each group was sampled in six replicates, and the treated and untreated cells were named CPTG and CPCG, respectively. Sample cells were fully ground in liquid nitrogen with 400 µL cooled methanol/acetonitrile/aqueous solution (4:4:2, v/v). After vortex oscillation mixing, the extracted samples were stored at −20°C for 60 min, and then centrifuged at 14,000 *g* for 20 min under 4°C (Heraeus Fresco 17, Thermo Fisher Scientific). Afterward the supernatant was recovered, and then evaporated in a vacuum rotary evaporator at 55°C until a constant weight was observed. The recovered extracts were re-dissolved in the acetonitrile aqueous solution (the ratio of acetonitrile to water was 1 to 1 (v/v)), followed by another centrifugation at 14,000 *g* for 15 min under 4°C. Thereafter, the collected supernatant was filtered through PTFE filters (0.22 μm) and then kept in an auto-sampler vial for the LC-MS/MS analysis (http://personalbio.bioon.com.cn/).

#### 2.8.2 Chromatographic conditions

All the detection was performed in a 1,290 series UHPLC (Dionex, CA, United States). Chromatographic separation was carried out in a ACQUITY UPLC BEH C18 column (100 mm × 2.1 mm, 1.7 μm, Waters, United States), and the column temperature was maintained at 40°C. The flow rate was typically set at 0.3 mL/min. The acidified water (0.1% formic acid (v/v), solvent A) and acetonitrile (solvent B) were used as mobile phases. The elution gradient was programmed as following: 0–0.5 min, 5% B; 0.5–1.0 min, 5% B; 1.0–9.0 min, 5–100% B; 9.0–12.0 min, 100% B; 12.0–15.0 min, 5% B. During the whole analysis process, the samples were placed in a 4°C automatic injector, and the volume of each injection was 5 µL (Benton et al., 2015).

#### 2.8.3 Mass spectrum conditions

The samples were separated by UHPLC and analyzed by q-exact-four-stage rod electrostatic field orbital trap high-resolution mass spectrometer (Thermo Fisher Scientific, United States). Electrospray ionization (ESI) mode of positive and negative ions were both adopted for the MS detection. MS detection conditions were as follows: ion source gas1 (gas1): 60, ion source gas2 (gas2): 60, curtain gas (cur): 30, source temperature: 320°C, ionsapary voltage floating (isvf) ± 3,500 V. Declustering potential (DP): ±60 V, collision energy: 35 ± 15 eV, exclude isotopes within 4 Da, candidate ions to monitor per cycle 6 (Julijana et al., 2013).

#### 2.8.4 Metabolomic data analysis

Qualitative and quantitative analyses were all carried out in Shanghai Personalbio Technology Co., Ltd. (http://personalbio.bioon.com.cn/). Multi-dimensional statistical analyses such as PCA (principal components analysis), orthogonal partial least squares discriminant analysis (OPLA -DA) were conducted. And single-dimensional statistical analysis like volcanic map was drawn by R software (https://www.r-project.org/).

#### 2.8.5 Differential metabolites analysis

The variable weight for the project (VIP) and fold change obtained by OPLA -DA model were combined to analyze and screen differential metabolites. VIP >1, *p*-value <0.05 and fold change >2 were used as criteria to screen the significantly changed metabolites (Luo et al., 2020).

### 2.9 Transcriptome detection and analysis

#### 2.9.1 Preparation of the sequencing samples

Cell samples were prepared according to the method described in Section 2.8.1. The treated and untreated cells were sampled in three replicates, which were named CPTG and CPCG, respectively. These samples were then sent to Shanghai Personalbio Technology Co., Ltd. for transcriptome detection.

#### 2.9.2 RNA extraction, cDNA library construction and high throughput sequencing (RNA-seq)

Total RNA was extracted from cells in a total RNA Extractor (SanGon, Shanghai, China) following the supplier’s instructions. The quality and quantity of extracted RNA were evaluated using agarose gel electrophoresis and 2,100 Bioanalyzer (Agilent, Germany). The quality-checked RNA was purified and then used as a template to synthesize cDNA first strand followed by cDNA second strand with Primscript™ First-Strand cDNA Synthesis Kit (Takara Bio, United States). PCR amplification was subsequently conducted to establish cDNA libraries. RNA-sequencing was performed on the IlluminaHiseq™ 2,500 platform (http://www.personalmedicine.cn/TechnicalPlatform) in Shanghai Personalbio Technology Co., Ltd.

#### 2.9.3 Processing of RNA-seq data and gene function annotation

The high-quality fragment reads (quality value ≥20) were spliced by software Trinity (software version number: 2.4.0) (Jonathan et al., 2022). All the assembled unigenes were searched against public protein databases, such as NCBI non-redundant protein sequences (NR) and Swiss-Prot for homology comparison. Only those matches with E-value below 10–5 were deemed significant (Park et al., 2021; Jonathan et al., 2022). Furthermore, gene function prediction and classification were assigned based on the database of the Gene Ontology (GO) and Eukaryotic Orthologous Groups of proteins (KOG). Moreover, secondary metabolic pathways were annotated using the Kyoto Encyclopedia of Genes and Genomes (KEGG) database.

#### 2.9.4 Differentially expressed genes (DEGs) analysis

DESeq was adopted to analyze DEGs with filtering conditions including |log_2_(Fold Change)| ≥ 1 and q-value <0.05. In order to analysis the metabolic and signal transduction pathways involved in DEGs, the DEGs enrichment of KEGG (https://www.genome.jp/kegg/genome) was performed by comparing DEGs with the entire background of all KEGG-associated unigenes with a hypergeometric test (*p* < 0.05).

### 2.10 QRT-PCR analysis

The DEGs expression levels were validated by QRT-PCR (quantitative real time polymerase chain reaction) assay. The RNA and cDNA required for this analysis were obtained according to the method in Section 2.9.2. and the primers were listed in Supplementary Table S3. Using the stable UBQ gene as the internal reference gene, QRT-PCR was performed using Ace-Q^®^ qPCR SYBR^®^ Green Master Mix (Vazyme). The reaction system was 20 μL, including 10 μL 2 × SYBR real-time PCR premixture, 1 μL cDNA and 0.8 μL primers. The qPCR procedures were as follows: initially denatured at 95°C for 5 min, followed by 40 cycles of 15 s at 95°C and 30 s at 60°C (Hazman, 2022). Gene relative expressions were calculated by 2^−ΔΔCT^ and geometric mean method (Zhao et al., 2020).

### 2.11 Integration analysis of transcriptome and metabolome

To reveal the inducement mechanism of the co-induction of 5-ALA and SA on the polyphenol biosynthesis, the differential metabolites and DEGs between the treated and untreated *C. paliurus* cells were analyzed through KEGG database (https://www.genome.jp/kegg/).

### 2.12 Statistical analysis

All the experiments were performed in triplicate and the results were given as the mean ± standard deviation (SD). The data was analyzed by DPS statistical software. Statistical analysis was performed with ANOVA followed by Duncan’s multiple range test (*p* ≤ 0.05).

## 3 Results

### 3.1 Effects of five elicitors on the cell growth and polyphenol biosynthesis

**FIGURE 1:**
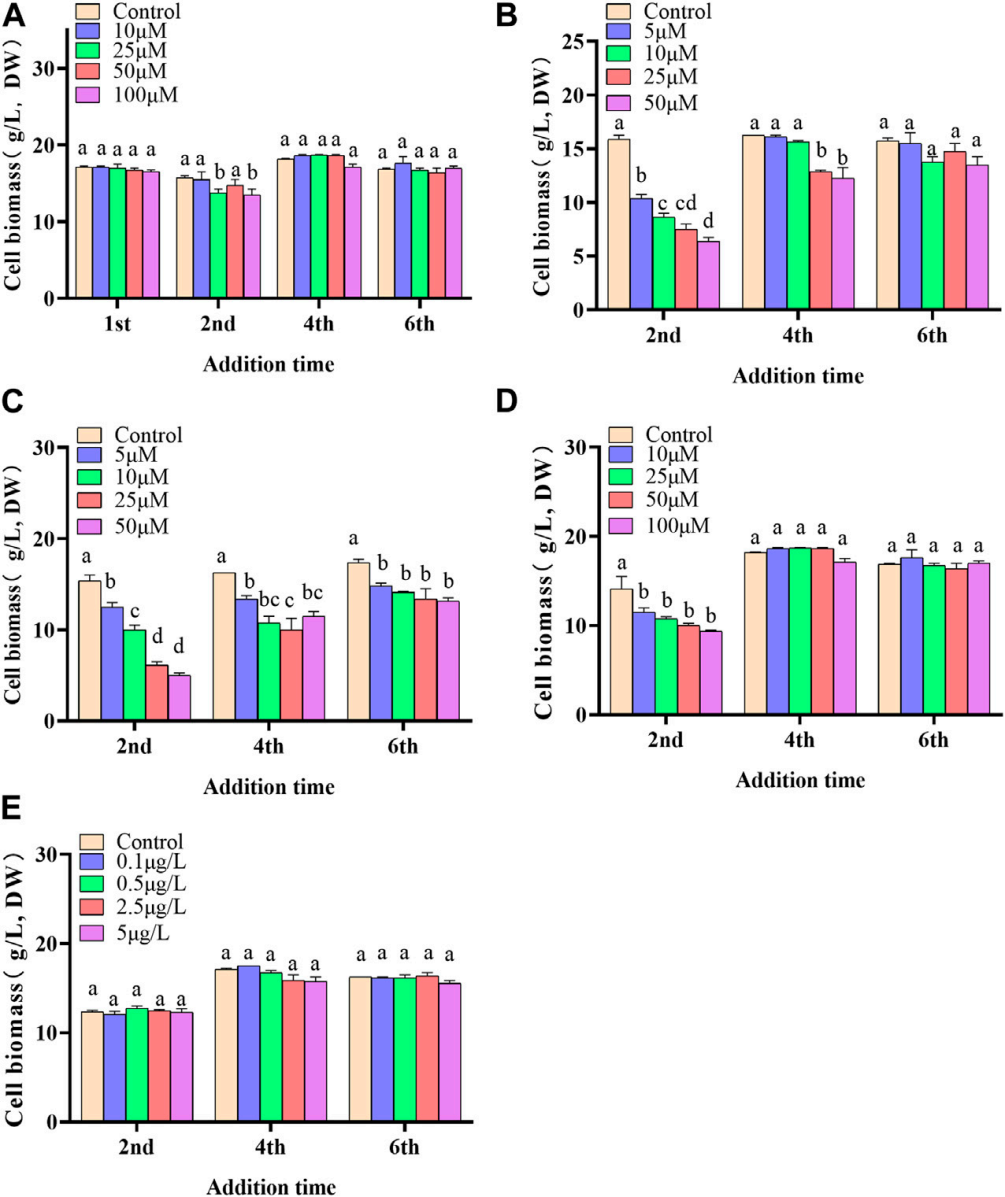
Effects of 5-ALA **(A)**, MeJA **(B)**, SNP **(C)**, SA **(D)**, ROE **(E)** on the biomass of the cultured *C. paliurus* cells.

**FIGURE 2:**
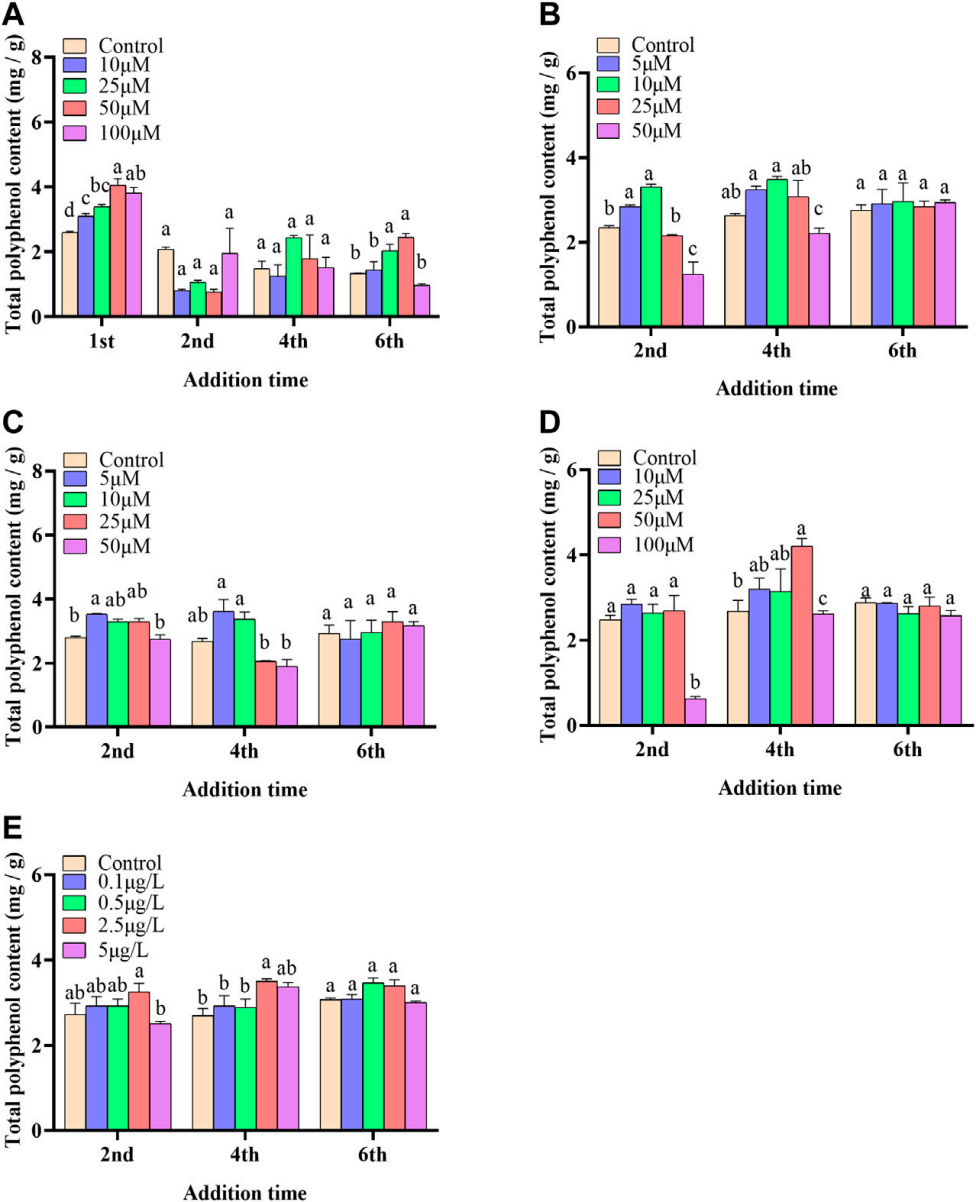
Effects of 5-ALA **(A)**, MeJA **(B)**, SNP **(C)**, SA **(D)**, ROE **(E)** on polyphenol content of the cultured *C. paliurus* cells.

**FIGURE 3:**
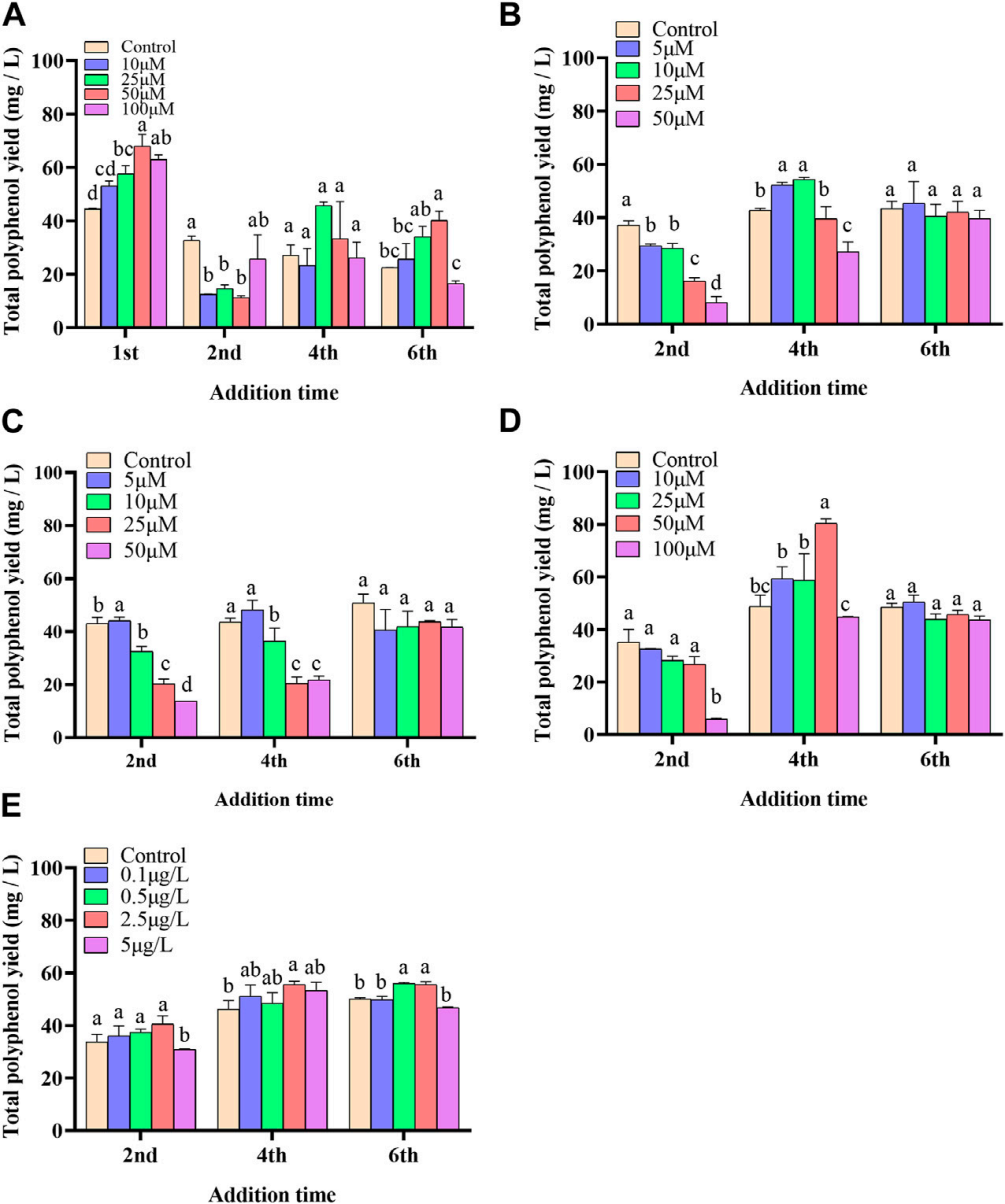
Effects of 5-ALA **(A)**, MeJA **(B)**, SNP **(C)**, SA **(D)**, ROE **(E)** on polyphenol yield of the cultured *C. paliurus* cells.

In the present paper, five elicitors including 5-ALA, SA, MeJA, SNP and ROE were used to improve the biosynthesis of polyphenols in the cultured *C. paliurus* cells. In terms of biomass (As shown in Figure 1), both 5-ALA and ROE showed no obvious effect on the growth of the cultured cells within the experimental concentration range, but MeJA displayed a negative effect, whenever they were added. Unlike above-mentioned three elicitors, SNP and SA hardly affected cell biomass when added on the 4^th^ and 6^th^ day while appeared adverse effect when added on the 2^nd^ day. With regard to polyphenol content and yield, all five elicitors showed promotion effects, but their increments differed from each other greatly (Figures 2, 3). The results showed that the promotion effect of the five elicitors on polyphenol content was in the order of SA, 5-ALA, SNP, ROE, and MeJA from best to worst, meanwhile SA and 5-ALA also displayed the most significant improvement on polyphenol yield of the cultured cells. It was recorded that when 50 μM 5-ALA was added on the 1^st^ day and 50 μM SA added on the 4^th^ day, the content reaching 4.05 and 4.3 mg/g, respectively, and the yield reaching 66.89 and 78.236 mg/L, respectively. Therefore, 5-ALA and SA were chosen for the further co-induction experiments.

### 3.2 Optimization of 5-ALA and SA concentration under co-induction

Comprehensive test was used to confirm whether the co-induction of 5-ALA and SA had a better induction effect and found the optimal concentration combination of 5-ALA and SA. The results suggested that the co-induction of 5-ALA and SA within the test concentration had no obvious effect on the biomass, but showed a significant influence on TPC and TPY (Supplementary Tables S4, S5). As shown in Supplementary Table S1, the highest content and yield appeared in A_2_B_2_, followed by A_2_B_1_ and A_1_B_1_. The highest TPC and TPY reached 7.945 mg/g and 129.213 mg/L, respectively. Multiple comparison results in Supplementary Table S6 indicated that both factor A (SA) and B (5-ALA) exhibited significant effect on TPC and TPY.

### 3.3 Comparison of the effects of 5-ALA, SA and the co-induction of 5-ALA and SA on the cell growth and polyphenol biosynthesis

As shown in Table 1, all of 5-ALA, SA and the co-induction of 5-ALA and SA had no obvious impact on the biomass, but showed a great promotion effect on the total polyphenol accumulation. With the addition of 50 μM 5-ALA at the beginning of cultivation, the yield of (+) - catechin, cyanidin-3-*O*-galactoside and procyanidin B1 rose from 31.74, 0.47 and 9.64 to 38.08, 3.04 and 27.11 mg/L, respectively, while with the addition of 50 μM SA on the 4^th^ day, the yield of (+) - catechin, cyanidin-3-*O*-galactoside and procyanidin B1 reached 49.28, 4.92 and 24.21 mg/L, respectively (Table 1). Relatively speaking, the co-induction of 5-ALA and SA had a much better improvement effect on polyphenol biosynthesis. Under the optimal co-induction parameter, the yields of (+) - catechin, cyanidin-3-*O*-galactoside and procyanidin B1 were 2.88, 28.83 and 4.33 times that of the control group, which were as high as 91.67, 13.55, and 41.83 mg/L, respectively. The above-mentioned results suggested that the co-induction of 5-ALA and SA could greatly promote the polyphenol biosynthesis of the cultured *C. paliurus* cells, and therefore have a promising application prospect.

**TABLE 1:** The content and yield of polyphenols in the cultured *C.paliurus* cells treated by the 5-ALA elicitation, SA elicitation, and the co-induction of 5-ALA and SA, respectively.

Compounds	Item	Blank group	5-ALA treatment	SA treatment	Co-induction of 5-ALA and SA
(+) - Catechin	Content (mg/g)	1.79 ± 0.73^b,c^	2.30 ± 0.08^b^	2.65 ± 0.11^b^	5.00 ± 0.22^a^
	Yield (mg/L)	31.74 ± 1.28^c^	38.08 ± 1.38b^b^	49.28 ± 2.1^b^	91.67 ± 4.16^a^
Procyanidin B1	Content (mg/g)	0.54 ± 0.01^c^	1.64 ± 0.13^b^	1.30 ± 0.30^b^	2.28 ± 0.30^a^
	Yield (mg/L)	9.64 ± 0.13^c^	27.11 ± 2.20^b^	24.21 ± 5.6^b^	41.83 ± 5.52^a^
Cyanidin 3-*O*-galactoside	Content (mg/g)	0.026 ± 0.02^c^	0.18 ± 0.01^b^	0.26 ± 0.16^b^	0.74 ± 0.12^a^
	Yield (mg/L)	0.47 ± 0.29^c^	3.04 ± 0.077^b^	4.92 ± 3.16^b^	13.55 ± 2.32^a^
Biomass (g/L, DW)		17.75 ± 0.56^a^	16.50 ± 0.06^a^	18.63 ± 1.20^a^	18.31 ± 0.16^a^
Total polyphenol content (mg/g)		2.35 ± 0.01^c^	4.05 ± 0.28^b^	4.2 ± 0.25^b^	8.00 ± 0.56^a^
Total polyphenol yield (mg/L)		41.05 ± 0.85^c^	66.89 ± 4.66^b^	78.23 ± 2.6^b^	147.12 ± 14.32^a^

^a^, ^b^ and ^c^ indicate the difference between groups at the 0.05 level. Differences between two groups are considered insignificant if the same letter exists, and significant if it does not exist.

Note: Significant difference analysis was performed with ANOVA, followed by Duncan’s multiple range test (*p* ≤ 0.05).

### 3.4 Metabolome analysis

**FIGURE 4:**
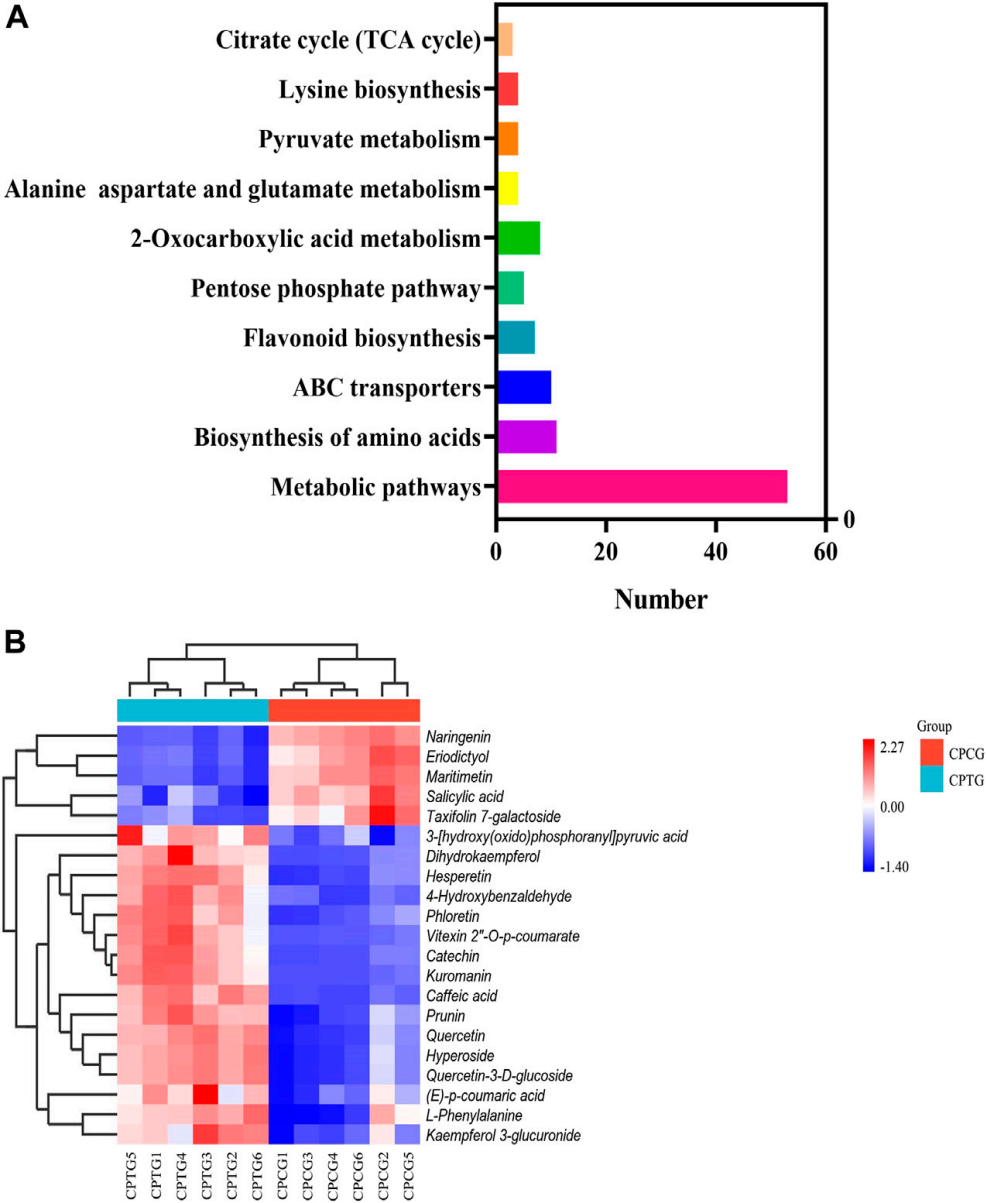
**(A)** Histogram of the top ten enriched biosynthetic pathways. **(B)** Cluster heat-map based on the analysis of 21 differential metabolisms in six samples.

To explore the effect of co-induction of 5-ALA and SA on the secondary metabolite synthesis in the cultured *C. paliurus* cells, especially polyphenols, the metabolome detection of the treated and untreated cells was performed using UPLC-MS/MS, which provided metabolic information for the interpretation of co-induction mechanism by dual omics analysis. A total of 970 compounds were found by UPLC-MS/MS determination with positive ion mode from the extracts of cell samples, including 138 upregulated metabolites and 39 downregulated metabolites in CPTG (Supplementary Figure S1A). Meanwhile, totally 958 compounds were identified under negative ion mode, among which 110 were upregulated and 34 were downregulated in the cells under the co-induction (Supplementary Figure S1B). Further analyses on the metabolite function and pathway were conducted to fully understand the metabolic changes caused by the co-induction. A total of 256 differential metabolites were enriched in 70 metabolic pathways using KEGG database (Figure 4A), including five significantly changed polyphenol biosynthesis pathways, and six samples were well clustered into CPCG and CPTG (Figure 4B). Furthermore, a total of 21 differential polyphenol metabolites were screened out from above-mentioned five pathways, of which 15 were upregulated and six were downregulated (Supplementary Table S7). For example, the content of vitexin 2″-*O*-p-coumarate, cyanidin-3-*O*-galactoside and catechin of CPTG were 25.10, 22.06 and 15.45 times that of CPCG, respectively (Supplementary Table S7). The above results and analyses once again indicated that the co-induction could greatly change the secondary metabolism and increase the polyphenol biosynthesis in the cultured *C. paliurus* cells.

### 3.5 Transcriptome sequencing results and analysis

#### 3.5.1 Functional annotation

**FIGURE 5:**
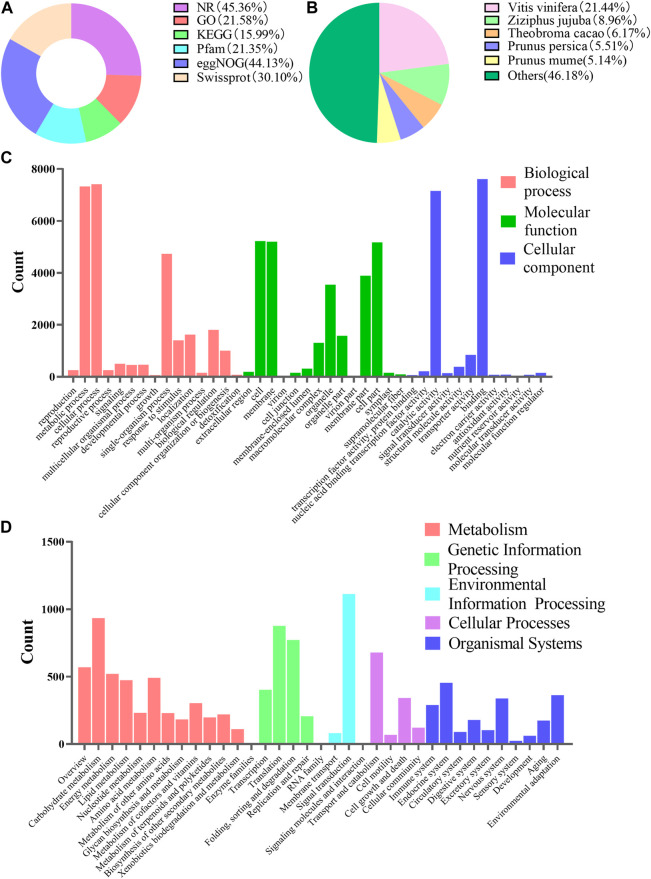
Functional annotations of unigenes of suspension-cultured *C. paliurus* cells. **(A)** Summary of unigenes annotation of *C. paliurus* suspension cell against six public databases. **(B)** Homologous species distribution of *C. paliurus* based on the annotation from NR database. **(C)** Histogram of unigene categories and secondary functional groups according to the GO annotation messages. **(D)** Unigene classifcation based on KEGG annotation.

In order to further understand the regulatory mechanism of co-induction on polyphenol biosynthesis in cells at the genetic level, we conducted transcriptome sequencing and analysis. Total RNA was extracted and sequenced. A total of 234,653,324 transcripts and 72,795,281 unigenes were generated by splicing and assembling using Trinity software (version number: 2.4.0) (Supplementary Table S8). All of the 72,795,281 unigenes (e-value) < 1 × E^−5^) were aligned with six public databases, and 45.36, 21.58, 15.99, 21.35, 44.13% and 30.10% of these unigenes had been annotated in NR, GO, KEGG, Pfam, eggNOG and Swiss-prot through BlastX, respectively (Figure 5A). It suggested that *Vitis vinifera* showed the highest homology with *C. paliurus* (21.44%) (Figure 5B). A total of 71,287 GO notes were divided into three categories such as Cellular Component (38.75%), Biological Process (37.62%) and Molecular Functions (23.62%) (Figure 5C). When blasting the unigenes of the sample cells against KEGG database, a total of 11,379 unigenes were annotated. The annotated information related to metabolic pathway was presented in Figure 5D.

#### 3.5.2 General analysis of DEGs

A total of 5,233 genes changed significantly under the co-induction of 5-ALA and SA (Supplementary Figure S2A). As shown in Supplementary Figure S2B, KEGG pathway enrichment analysis showed that the expression of structure genes of the pathways related to the polyphenol biosynthesis changed notably, including “Phenylpropanoid biosynthesis,” “Flavonoid biosynthesis,” and “Phenylalanine metabolism” with 30, 5 and 7 DEGs, respectively. Meanwhile, other pathways like signal transduction pathways such as MAPK signaling pathway-plant (15DEGs), Linoleic acid metabolism (4DEGs) and Plant hormone signal transduction (23DEGs) were also involved in the co-induction of 5-ALA and SA (Supplementary Figure S2B). In conclusion, the improvement of polyphenol biosynthesis could be attributed to the DEGs of polyphenol biosynthesis pathway under the co-induction. Therefore, the differentially expressed transcription factors (DETFs) and structural genes related to polyphenol biosynthesis pathway were further explored in detail.

#### 3.5.3 Analysis of DETFs related to polyphenol biosynthesis

**FIGURE 6:**
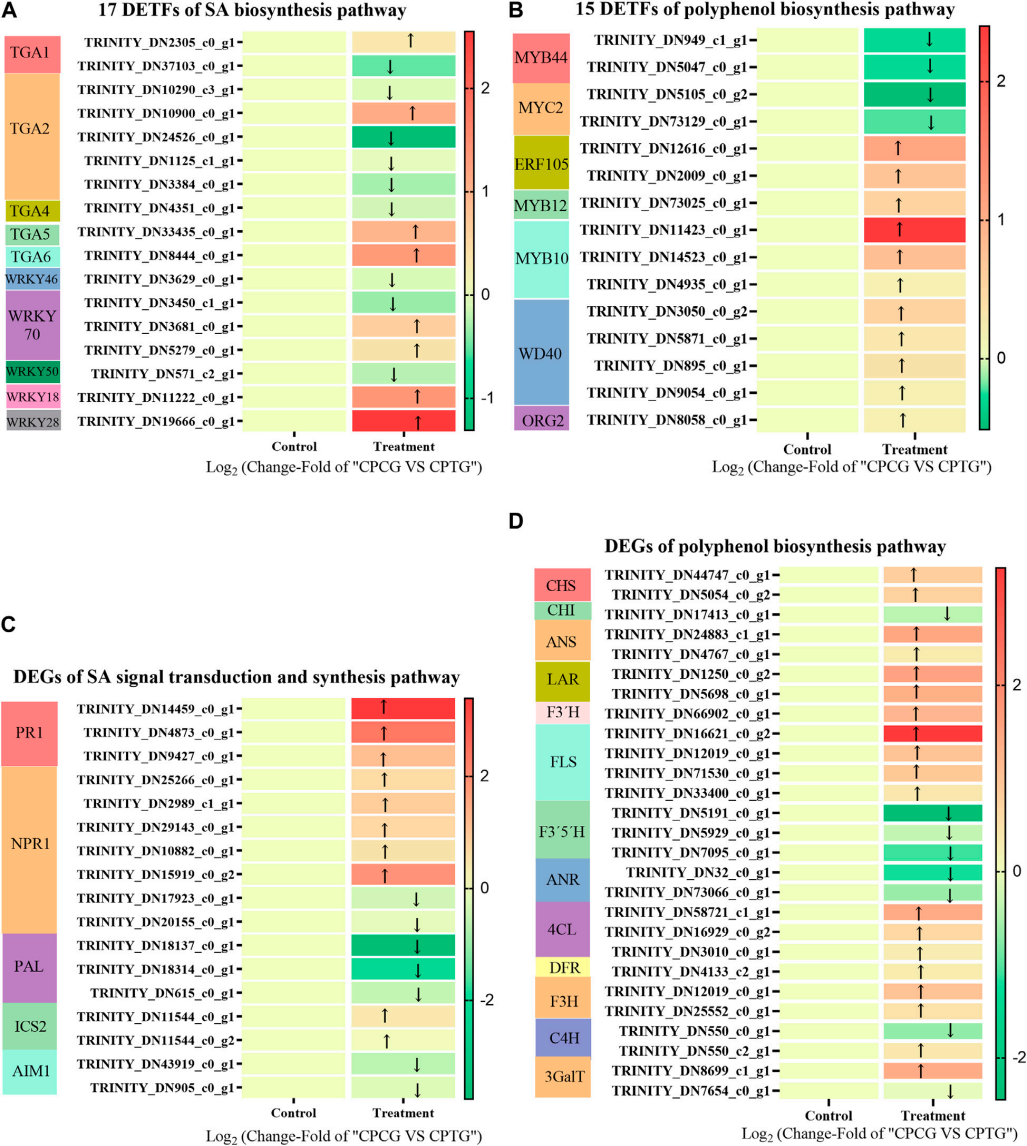
The DETFs and DEGs in suspension-cultured *C. paliurus* cells under the co-induction of 5-ALA and SA. **(A)** 17 DETFs related to SA signal transduction and biosynthesis pathway in the cultured *C. paliurus* cells under the co-induction of 5-ALA and SA. **(B)** 15 DETFs related to polyphenol biosynthesis pathway in the cultured *C. paliurus* cells under the co-induction of 5-ALA and SA. **(C)** DEGs related to SA signal transduction and biosynthesis pathway in the cultured *C. paliurus* cells under the co-induction of 5-ALA and SA. **(D)** DEGs related to polyphenol biosynthesis pathway in the cultured *C. paliurus* cells under the co-induction of 5-ALA and SA.

A total of 7,351 DETFs were identified between CPCG and CPTG (Supplementary Figure S3), among which 17 were involved in SA bio-synthetic pathway (Figure 6A), and 15 were related to polyphenol biosynthesis pathway (Figure 6B). Further analysis revealed that the expression of two *CpMYB44s*, one *CpMYB12*, two *CpERF105s*, three *CpMYB10s*, four *CpWD40s*, one *CpORG2* and two *CpMYC2s* changed significantly under the co-induction of 5-ALA and SA. Among these significantly changed DETFs, *CpMYB10* and *CpERF105* showed a remarkable upregulation expression, on the contrary *CpMYB44* and *CpMYC2* presented a downregulation expression. TGA family is an important regulator of the signal transduction pathway related to SA biosynthesis. Results indicated that *CpTGA1*, *CpTGA2*, *CpTGA4*, and *CpMYC2* were all downregulated under co-induction, among which *CpTGA1* and *CpTGA2* decreased significantly, while *CpWRKY18*, *CpWRKY28*, *CpWRKY70*, and *CpTGA6* were upregulated, especially *CpWRKY28* (Figure 6A). Based on our determination results and the above analyses, it could be deduced that the co-induction of 5-ALA and SA might improve polyphenols accumulation by changing the expression of TFs related to polyphenol biosynthesis.

#### 3.5.4 Analysis of DEGs related to polyphenol biosynthesis

As shown in Figure 6C, three *CpPALs*, two *CpICS2s* (isochorismate synthase 2) and two *CpAIM1s* (recombinant Absent In Melanoma 1) were annotated in SA biosynthesis pathway, meanwhile seven *CpNPR1* genes (non-expresser of PR genes 1) and three *CpPR1* genes (PR genes 1) were annotated in SA signal transduction pathway (as shown in Figure 6C). Under the co-induction of 5-ALA and SA, the expressions of two annotated *CpPALs* were significantly downregulated, while the expressions of all other annotated SA synthesis pathway genes did not show significant changes, which suggested that co-induction might inhibit SA synthesis by suppressing the expression of *CpPAL*. We speculated that the exogenous addition of 50 μM SA downregulated the expression of *CpPAL*, a key gene for SA endogenous biosynthesis, through feedback inhibition during co-induction. Furthermore, the exogenously added SA markedly enhanced the expressions of three annotated *CpPR1s* and one annotated *CpNPR1* (Figure 6C), which made the SA signaling pathway more active, and eventually activated the polyphenol synthesis pathway. As shown in Figures 6A, D total of nine upregulated enzyme genes and five downregulated enzyme genes of the polyphenol biosynthesis pathway were found between CPCG and CPTG. Under the co-induction of 5-ALA and SA, the expressions of *CpANS*, *CpLAR*, *CpF3′H*, *CpFLS*, *Cp4CL*, *CpF3H,* and *Cp3GalT* were significantly increased, meanwhile the expressions of *CpPAL*, *CpF3′5′H,* and *CpANR* genes were significantly decreased (Figure 6D), which led to a notable improvement of polyphenol synthesis.

### 3.6 QRT-PCR verification of DEGs

**FIGURE 7:**
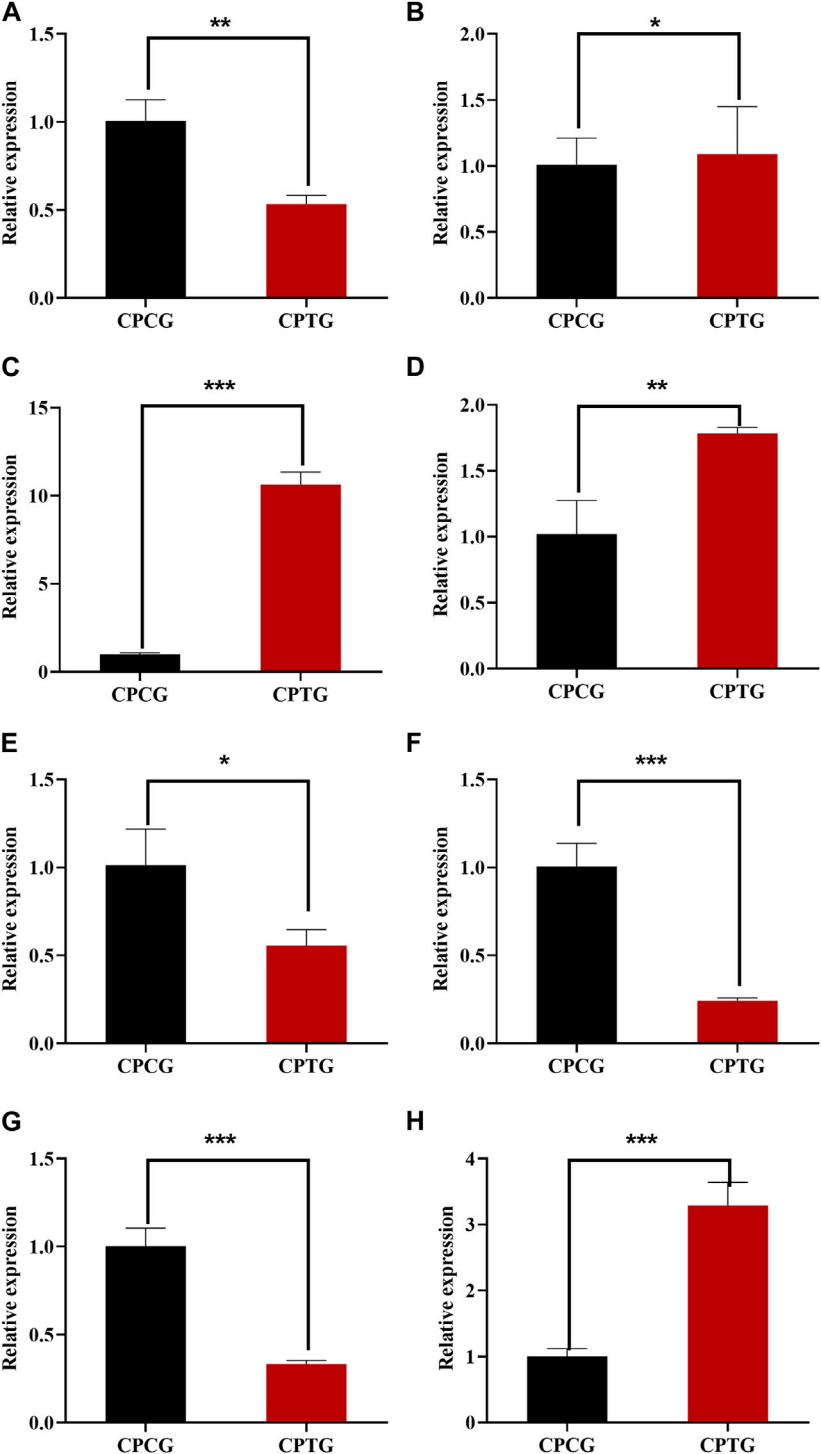
QRT-PCR determination results of 8 DEGs between CPCG and CPTG. **(A)** *CpTGA2* relative expression. **(B)** *CpNPR1* relative expression. **(C)** *CpPR1* relative expression. **(D)** *CpTGA6* relative expression. **(E)** *CpFLS* relative expression. **(F)** *CpF3′5′H* relative expression. **(G)** *CpANR* relative expression. **(H)** *CpLAR* relative expression. Note: Significant difference analysis was conducted by independent samples *t*-test with a *p*-value of at least less than 0.05.

The expressions of the identified 8 DEGs (*LAR*, *FLS*, *F3′5′H*, *ANR*, *TGA2*, *TGA6*, *NPR1*, and *PR1*) were further verified by QRT-PCR. The determination results (as shown in Figure 7) suggested that the expressions of all candidate genes were consistent with the transcriptome data except *FLS*, indicating that our RNA-seq detection and analysis were relatively reliable.

### 3.7 Interpretation of co-induction mechanism by integrated analysis of transcriptome and metabolome

**FIGURE 8:**
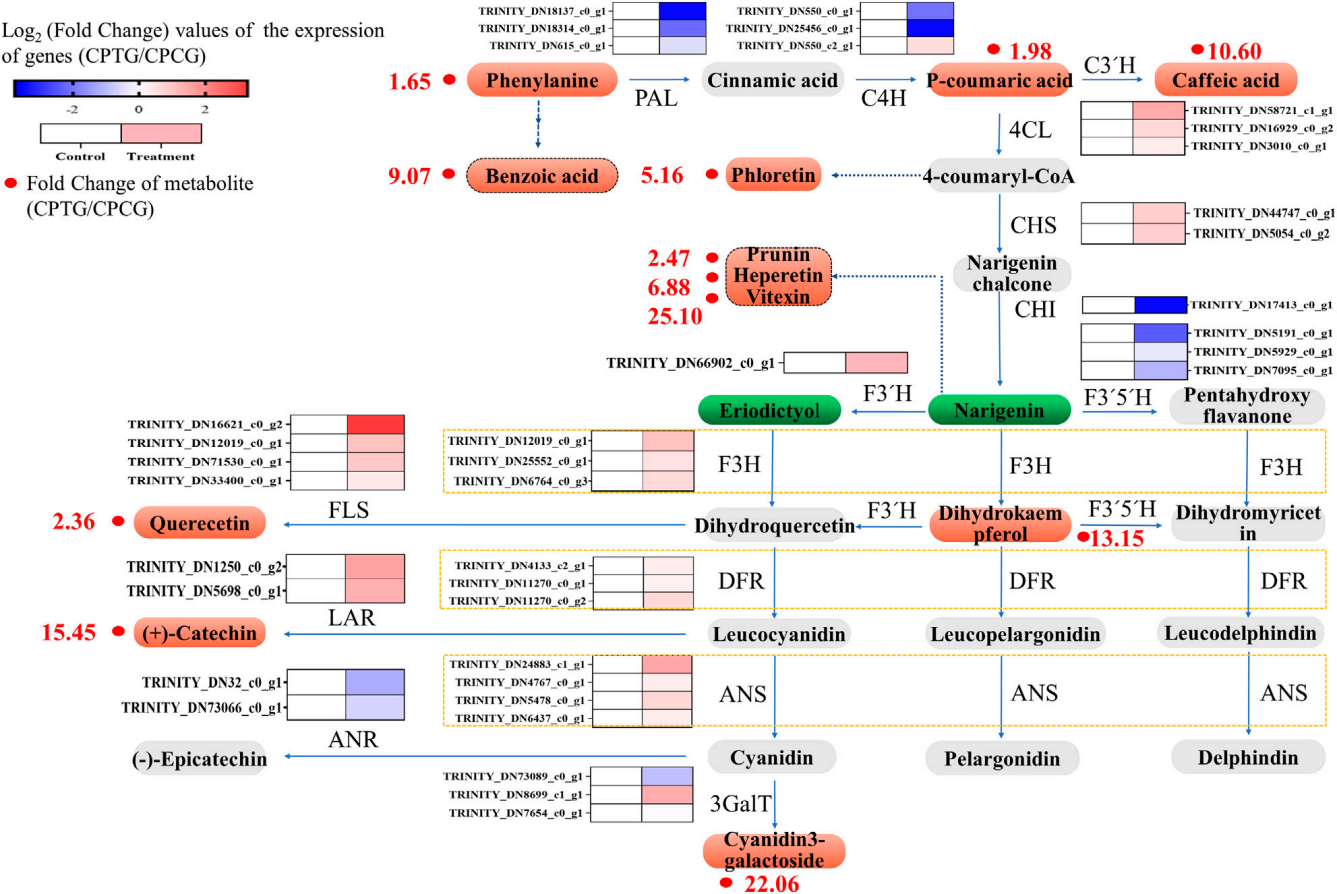
Integration analysis figure of transcriptome and metabolome of the polyphenol biosynthesis in the *C. Paliurus* cells under the co-induction of 5-ALA and SA.

According to the above transcriptome analysis, we found that the expression of most upstream key structural genes in the polyphenol biosynthesis pathway were downregulated, while the expressions of downstream key structural genes were overall upregulated (Figure 8). It could be speculated that the upstream genes in the polyphenol biosynthesis pathway firstly responded to the stimulation at the beginning of co-induction and led to the accumulation of the upstream metabolites, afterwards the expression of midstream and downstream genes upregulated successively, and finally resulted in the improvement of the content and yield of polyphenols in the treated cells. Because our samples for RNA-seq were collected on the 6^th^ day (i.e., 2 days after the SA addition and 6 days after 5-ALA addition), at this moment the sample cells were in the middle and late stage of the co-induction, in which the expression of upstream genes such as *CpPAL*, *CpC4H*, and *CpCHI* had already retreated, and the midstream and downstream genes like *CpANS*, *CpLAR*, *CpF3′H*, *CpFLS*, *Cp4CL*, *CpF3H*, and *Cp3GalT* were highly expressed (Figure 8), and therefore polyphenol accumulations were significantly enhanced. Consequently, our metabolome data showed that the content of dihydrokaempferol, quercetin, quercetin-3β-D-glucoside, cyanidin-3-*O*-galactoside and catechin in the elicited cells were significantly upregulated, whose content were 13.15, 2.36, 15.45, 22.06, and 2.21 times higher than those in the control group, respectively (Supplementary Table S7).

Due to the increase of metabolic flow along the whole pathway, other polyphenol substances in the bypasses of polyphenol biosynthesis pathway were also significantly increased accordingly. For example, the content of benzoic and phloretin derived from phenylalanine and 4-coumaryl were greatly raised, which were 9.07 and 5.16 times that in the untreated cells, respectively (Supplementary Table S7). Besides, the content of prunin, hesperetin, vitexin 2″-*O*-p-coumarate converted from naringin were 1.47, 5.88, 24.10 times higher than those in the control group, respectively, which might also be one of the reasons for the reduce of naringin and eriodictyol (Supplementary Table S7).

In summary, the transcriptome data were generally consistent with the metabolome analysis. The co-induction stimulated the expression of key genes related to polyphenol biosynthesis pathway, and finally promoted the accumulation of polyphenols in the cultured cells (as shown in Supplementary Figure S4).

## 4 Discussion

Elicitation is generally regarded as one of the most effective ways to stimulate plant secondary metabolite synthesis. In the present research, five elicitors such as 5-ALA, SA, SNP, MeJA, and ROE were chosen to improve polyphenol biosynthesis in our cultured *C. paliurus* cells. We found that there were significant different promotion effects among the five elicitors, meanwhile both addition time and concentration also showed a strong influence on the polyphenol synthesis. Firstly, all the selected five elicitors obviously improved the TPC and TPY of the cultured cells, among which 5-ALA and SA were much more effective in comparison. TPY of the cultured cells stimulated by SA and 5-ALA reached 78.23 mg/L and 66.89 mg/L, respectively. We speculate that different elicitors have different physic-chemical and biological properties, and therefore show different promotion effects in our cultured *C. paliurus* cells. According to Yu et al. (2019), SA, NaCl, and AgNO_3_ exerted different influences on primary metabolites and secondary metabolites synthesis in the suspension culture of *Salvia miltiorrhiza* Bunge cells. Secondly, addition time plays an important role in the elicitation. Our results suggested that 5-ALA showed a stronger promotion effect when added on the 1^st^ day, while SA worked better when added on the 4^th^ day, which led to a TPY increment of 63% and 90% in our cultured cells, respectively. Li et al. (2021) also found that rosmarinic acid yield basically remained unchanged when 200 μM SA was added on the 12^th^ day (cells in plateau phase), while increased 3.3 times when added on the 10th day (cells in mid-exponential phase) in suspension culture of *Origanum vulgare* cells. Consequently, we deduce that cells in different culture phase have different physiological states, and therefore might present different responses to the external stimulus. Thirdly, elicitor concentration has a profound effect on the induction, which may act on cell growth or on the regulation of polyphenol synthesis. Our experiment data indicated that both 50 μM 5-ALA and SA showed the best stimulatory effect on polyphenol synthesis in the series of our test concentrations. Similar results had been reported by Kara et al. (2019). In addition, our results showed that MeJA with a concentration more than 10 μM had an adverse effect on polyphenol synthesis, however, Liu et al. found that MeJA at a concentration of 200 μM enhanced the biosynthesis of chlorogenic acid and its derivatives in *Gardenia jasminoides* Ellis cells (Kara et al., 2019), suggesting that elicitors might display species-specific and metabolite-dependent stimulatory effects in plant secondary metabolite synthesis.

To further enhance the synthesis of polyphenols in the cultured cells, a co-induction technology of 5-ALA and SA were developed. With the developed co-induction technology, TPC and TPY were 2.4 and 2.58 times than those of the control group, respectively. It is interesting to note that the content of cyanidin-3-*O*-galactoside, catechin, dihydrokaempferol and caffeic acid in the co-induced cells increased 21.06, 14.45, 12.15, and 9.60 times. The search for synergistic inducers and the establishment of co-induction techniques to obtain high yields of plant secondary metabolites is now a novel technique that has attracted the attention of many scholars. Li et al. (2021) found a synergistic effect between Phe and SA and accordingly established a co-induction technology for *O. vulgare* cells to obtain a high yield of polyphenols. Under the developed co-induction technology, the polyphenol yield reached 752.93 mg/L, which was respectively 2.32 and 1.13 times that of Phe and SA elicitation individually. According to the report of Zheng et al., 5-ALA and 24-EBL significantly stimulated anthocyanin accumulation in the cultured apple callus, however the promotion effect of co-induction was much stronger than that of 5-ALA and 24-EBL separately (Zheng et al., 2018). With the optimized co-induction technique, a 28.51-fold increase of anthocyanin content was obtained. From the reports available so far, although there have been many studies on co-induction to promote the synthesis of secondary metabolites in plants, the mechanisms of induction signaling and regulation are not yet well understood, which need to be further explored.

In the present paper, integration analysis of transcriptome and metabolome was conducted to reveal the promotion mechanism of 5-ALA and SA co-induction on polyphenol synthesis in cultured *C. paliurus* cells. The results showed that the expression of *CpPAL* in the treated cells were significantly downregulated, while *CpPR1* and *CpNPR1* were significantly upregulated. Zhang and Li (2019) had already demonstrated that there are mainly two pathways to bio-synthesize SA in plants, namely, iso-branched acid pathway and shikimic acid pathway. PAL was a key structural gene in shikimic acid pathway. An increase in SA concentration can inhibit PAL gene expression through negative feedback. Therefore, we deduced that exogenous addition of SA inactivated the shikimic acid pathway by suppressing PAL gene expression, which caused the inhibition of SA synthesis in the co-induced cells. PR1 and NPR1 were two critical genes in SA signal transduction pathway (Saleh et al., 2015). The upregulation of *CpPR1* and *CpNPR1* expression suggests that co-induction of 5-ALA and SA activated the SA signaling pathway. We inferred that the exogenous addition of SA led to an increase in intracellular SA concentration, which further caused a stress response and consequently activated the SA signaling pathway. Previously, it had been reported that exogenous addition of SA could promote the expression of PR1 gene in *Solanum lycopersicum* (Bozbuga, 2020). In addition, researches had validated that SA could affected the transcriptional activity of NPR1 through various protein modifications, thereby regulating downstream gene expression (Saleh et al., 2015; Withers and Dong, 2016). When SA concentration in plants is low, NPR1 forms multimers and locates in the cytoplasm, meanwhile serine at positions 55 and 59 of NPR1 are phosphorylated, which results in the inhibition of transcriptional activation of NPR1 (Withers and Dong, 2016). When the concentration of SA rises to a certain level, NPR1 changes from a multimer to a monomer and transfers to the nucleus. In the nucleus, NPR1 is ubiquitinated and phosphorylated at positions 11 and 15, which further enhance the transcriptional activation of NPR1 and promote the expression of SA downstream gene (Withers and Dong, 2016).

DETFs and DEGs analysis are commonly used to interpret the mechanisms of secondary metabolic regulation in plants. Our transcriptome sequencing data showed that the expression of TFs such as *CpMYB10*, *CpERF105*, and *CpWRKY28* in the co-induced cells were significantly upregulated, while the expression of *CpMYB44* was downregulated (Figure 6B). In addition, the expression of structure genes in polyphenol synthesis pathway, including *CpANS*, *CpLAR*, *CpF3′H*, *CpFLS*, *Cp4CL*, *CpF3H*, and *Cp3GalT*, were notably increased. It has been confirmed that MYB, bHLH, ERF were the critical TFs regulating the polyphenol biosynthesis pathway in most plants, especially phenylalanine metabolic pathway (Blanco et al., 2013; Enrique et al., 2017). MYB44 was reported an important negative regulator in the regulation of anthocyanin synthesis. According to Liu et al. (2019), *StMYB44* repressed anthocyanin accumulation in leaves of N. tabacum by suppressing the activity of DFR promoter. Lin-Wang et al. (2014) reported that *FvMYB10* co-expressed with *FvbHLH33* strongly activated the *AtDFR*, *FvDFR*, and *FvUFGT* promoters, which further significantly promoted the anthocyanin biosynthesis in strawberry (*Fragaria vesca*). When treated by low temperature, ERF105 expression was positively correlated with the accumulation of polyphenols in *Arabidopsis thaliana* (Bolt et al., 2017). Therefore, we could conclude that co-induction promoted the expression of key genes such as *CpDFR* in the anthocyanin synthesis pathway by down-regulating *CpMYB44* expression and up-regulating *CpMYB10* and *CpERF105* expression, thus activated the anthocyanin metabolic pathway and ultimately significantly enhanced anthocyanin synthesis in the cultured red *C. paliurus* cells. In addition, based on our results of integration detection and analysis of transcriptome and metabolome, it could be inferred that co-induction significantly increased the expression levels of *CpF3H*, *CpFLS*, and *CpLAR*, thereby led to a 12.15-, 1.46-, and 14.45-fold increase in the synthesis of dihydromyricetin, quercetin and chitosan, respectively.

The lignin synthesis pathway is also an important branch of the phenylpropane metabolic pathway (Supplementary Figure S5), which is closely related to the flavonoid synthesis pathway, and its expression status may affect the synthesis of flavonoids and anthocyanins, which in turn influence the accumulation of polyphenols. From our experimental results, lignin synthesis was inhibited under co-induction of 5-ALA and SA. As shown in Supplementary Figure S6, the expression levels of *CpMYB1*, *CpMYB6*, *CpMYB46*, *CpMYB58*, and *CpMYB63* were decreased, especially *CpMYB1* and *CpMYB63* were significantly decreased, while *CpMYB31* was upregulated, which may lead to a decrease in the expression levels of downstream structural genes such as *CpCCoMT*, *CpCOMT*, *CpCCR*, and *CpCAD*. It was reported that *AtMYB58* and *AtMYB63* are the first identified true lignin-specific transcript factors in *Arabidopsis*, and act as activators that regulate most monolignol genes to synthesize lignin (Perkins et al., 2020). Fornalé et al. (2006) found that the over-expression of *ZmMYB31* may affect the expression of COMT gene and produced a decrease in lignin content in *Zea mays*. These changes in the expression levels of genes ultimately inhibited the entire lignin biosynthesis pathway. Therefore, we could deduce that co-induction may reduce the metabolic flow of the phenanthrene metabolic pathway to the bypass in order to promote the flavonoid and anthocyanin biosynthesis pathways, and eventually enhance the accumulation of polyphenols.

At present, there are two major obstacles for industrial production of secondary metabolites by plant cell culture, i.e., low yields and high costs. As a result, only a few plant secondary metabolites have been successfully industrialized by plant cell culture, which are either of high value or of high content in the cultured plant cells. Frankly speaking, polyphenol content of the cells is not much high, so in order to create a foundation for large-scale production in the future, it is necessary to study the technical means to increase its content. However, the polyphenol yield of our cultured *C. paliurus* cells was 147.12 mg/L under the co-induction of 5-ALA and SA, which is currently not enough to meet the requirements of industrialized production. Consequently, we will consider the following three ways to further improve polyphenol yield in our future researches. Firstly, cell lines with high polyphenol content can be obtained through mutagenesis and screening. Secondly, screening novel inducers is a feasible approach. Thirdly, optimization of the co-induction technology is another potential way.

However, the production of polyphenols by our red *C. paliurus* cell culture had the outstanding advantage of a relatively short production cycle, which was only 6 days. Currently, most production cycles for secondary metabolite production by plant cell culture are about 10–15 days (Ten Hoopen et al., 1994; Liu et al., 2020). For example, the production cycle of paclitaxel with Taxus suspension cells was 15 days (Li et al., 2009). Relatively speaking, the production of polyphenols by red *C. paliurus* cell culture had a shorter cycle, which could reduce the possibility of contamination and lower production costs to a certain extent.

## 5 Conclusion

Five elicitors were used to stimulate the polyphenol synthesis in the cultured *C. paliurus* cells. 5-ALA and SA showed better promotion effects and therefore were selected to develop a co-induction technology. Under the optimized co-induction technology (50 μM 5-ALA and 50 μM SA were added on the 1^st^ and 4^th^ day after inoculation, respectively), the content and yield of total polyphenols in the treated cells reached 8.0 mg/g and 147.12 mg/L, respectively. (+)-Catechin was the most abundant polyphenol, whose content and yield were as high as 5 mg/g and 91.67 mg/L, respectively. Integration detection and analysis of transcriptome and metabolome showed that the co-induction of 5-ALA and SA significantly changed the expressions of 14 pathway genes and 7 TFs related to polyphenol biosynthesis. Under the co-induction, the expressions of TFs such as *CpERF105*, *CpMYB10*, and *CpWRKY28* increased significantly, which might further upregulated the expressions of *CpANS*, *CpLAR*, *CpF3′H*, *CpFLS*, *Cp4CL*, *CpF3H*, and *Cp3GalT* and downregulated the expressions of *CpPAL*, *CpANR*, and *CpF3′5′H*, and finally enhanced the polyphenol accumulation.

## Data Availability

The datasets presented in this study can be found in online repositories. The names of the repository/repositories and accession number(s) can be found in the article/Supplementary Material.
