# Supplementary material for: Investigating the induction of polyphenol biosynthesis in the cultured Cycolocarya paliurus cells and the stimulatory mechanism of co-induction with 5-aminolevulinic acid and salicylic acid
Source: Front Bioeng Biotechnol. 2023 Mar 9;11:1150842. doi: 10.3389/fbioe.2023.1150842 (PMC10034720; doi:10.3389/fbioe.2023.1150842)
Supplement: Supplementary file 1 [file DataSheet1.docx]

**Supplementary material**

**Fig S1**


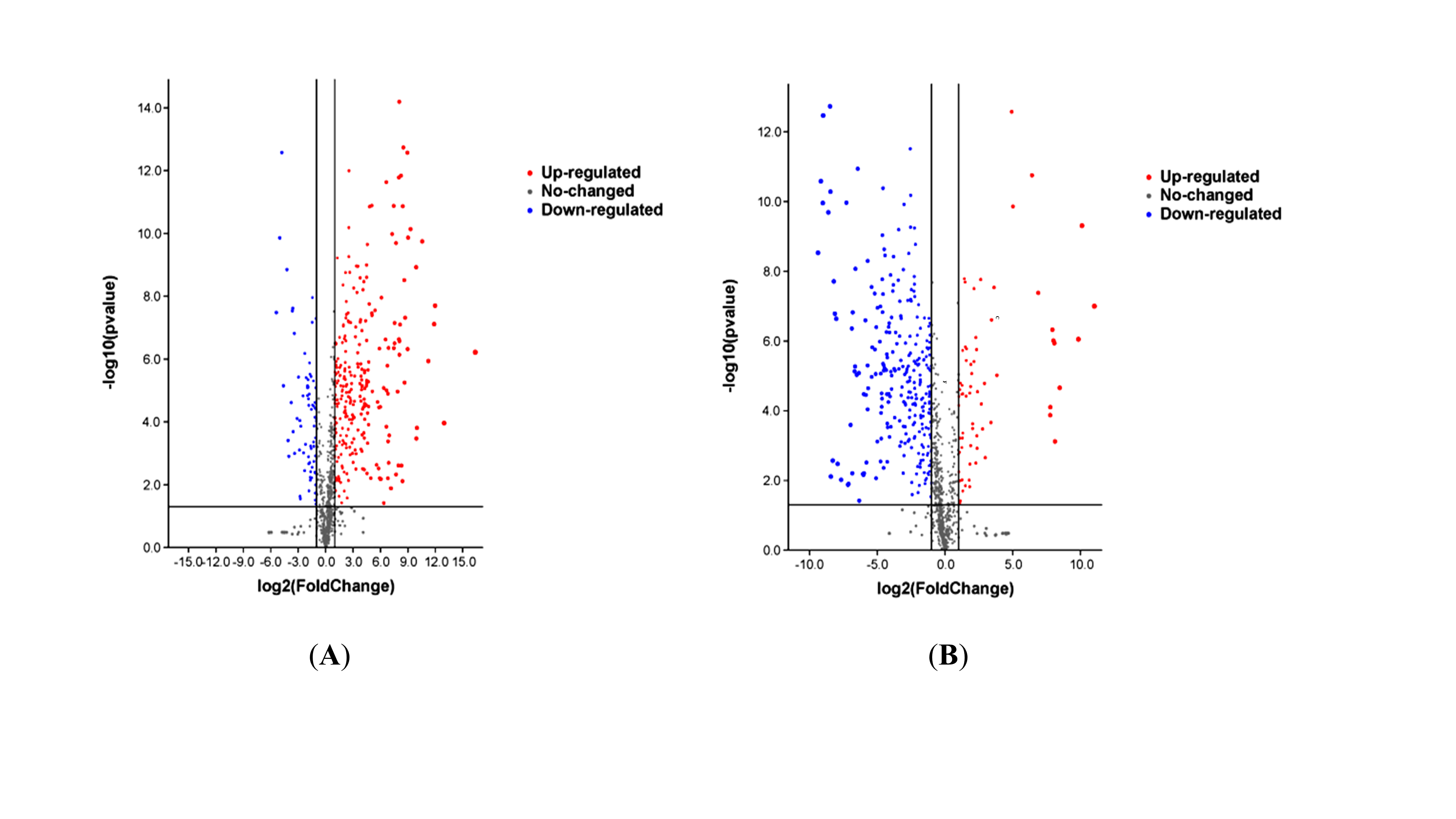


**Fig S1.** (**A**) Volcano maps for differential metabolites（positive ion mode). (**B**) Volcano maps for differential metabolites (negative ion mode).

**Fig. S2**

**
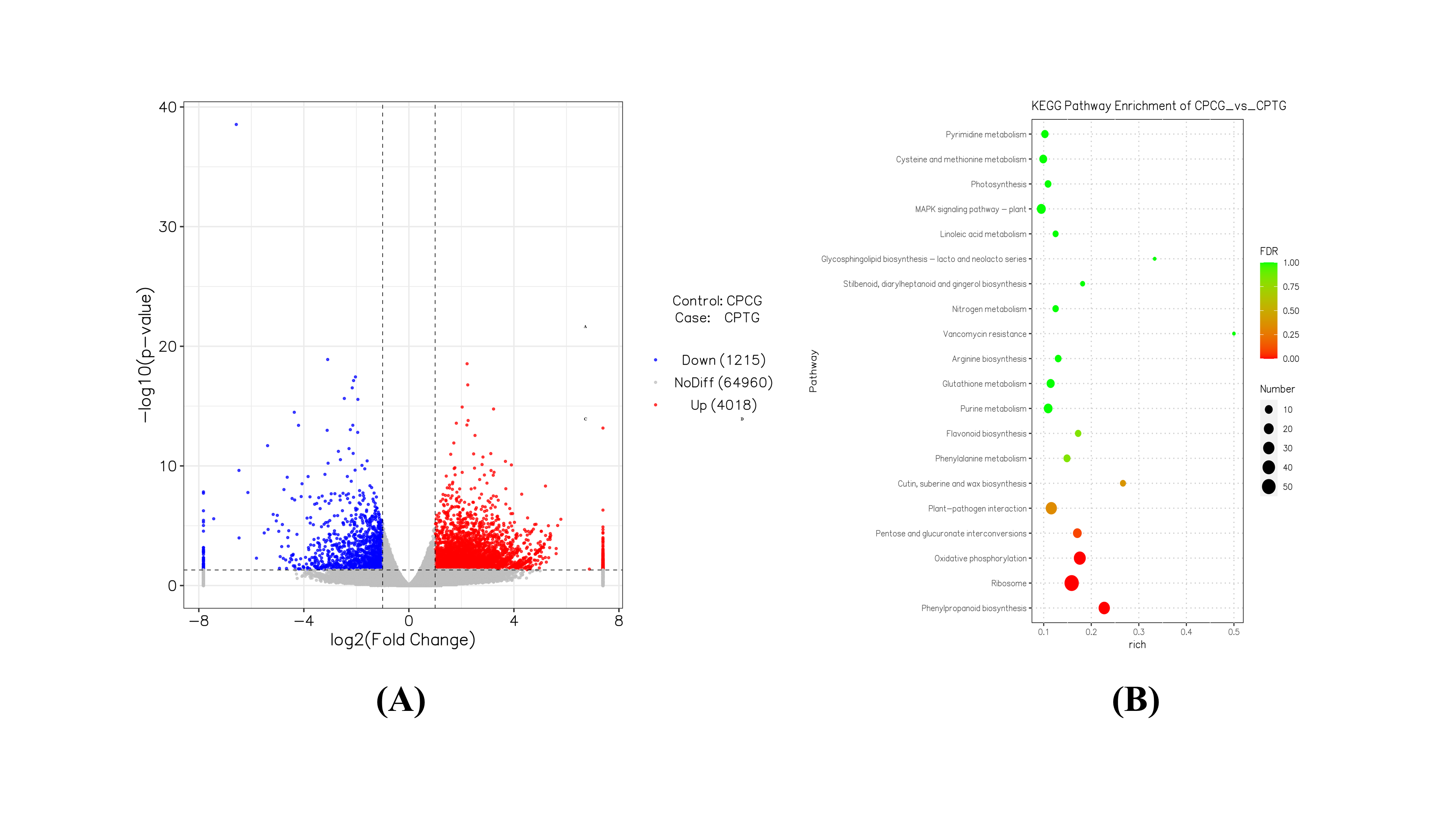
**

**Fig S2.** (**A**) The volcanic map of DEGs. (**B**) KEGG pathway enrichment of CPCG_CTCG.

**Fig. S3**


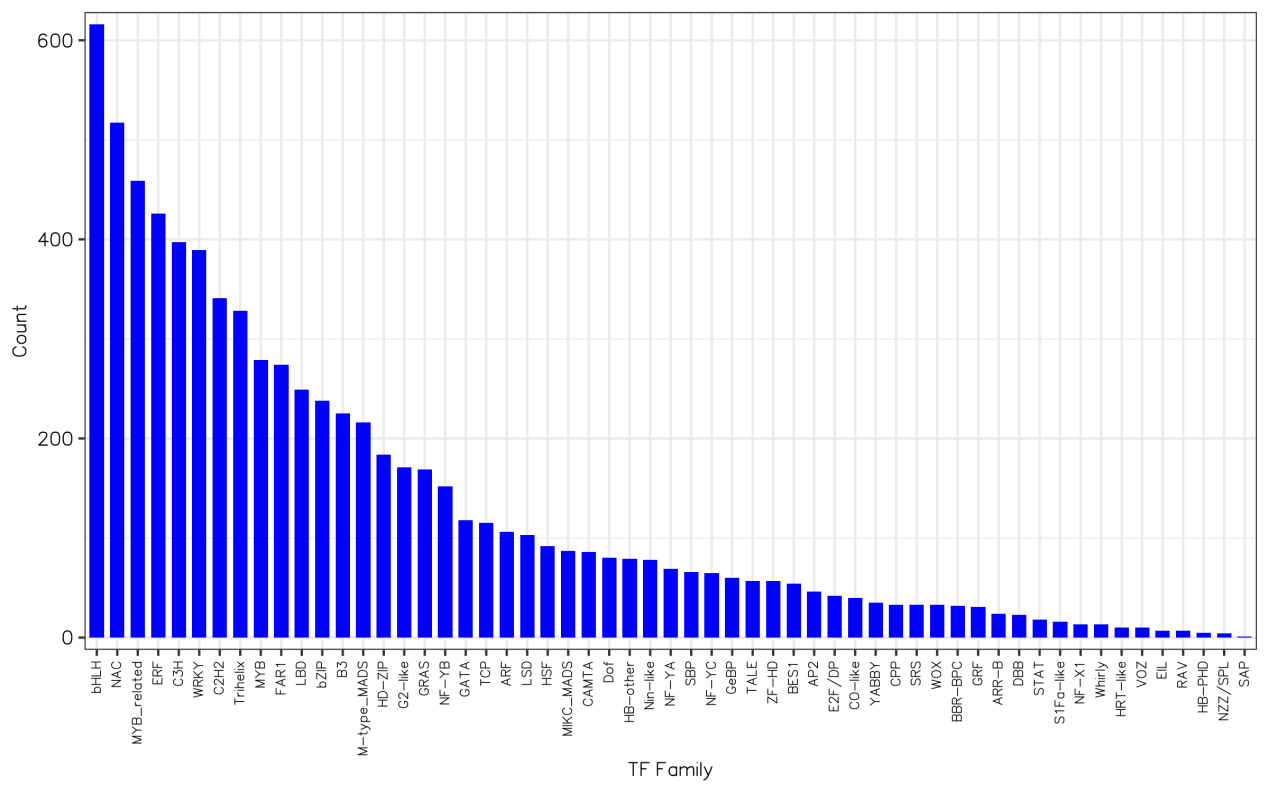


**Fig S3.** A total of 7351 identified DETFs between CPCG and CPTG.

**Fig. S4**

**Co-induction of 5-ALA and SA**

15 DETFs related to polyphenol biosynthesis pathway

14 DEGs related to polyphenol biosynthesis pathway

10 significantly increased polyphenol compounds

5-ALA

SA

**Fig S4.** A regulatory mechanism sketch of the co-induction of 5-ALA and SA on polyphenol biosynthesis by changing the expression of 15 DETFs and 14 DEGs.

**Fig S5**


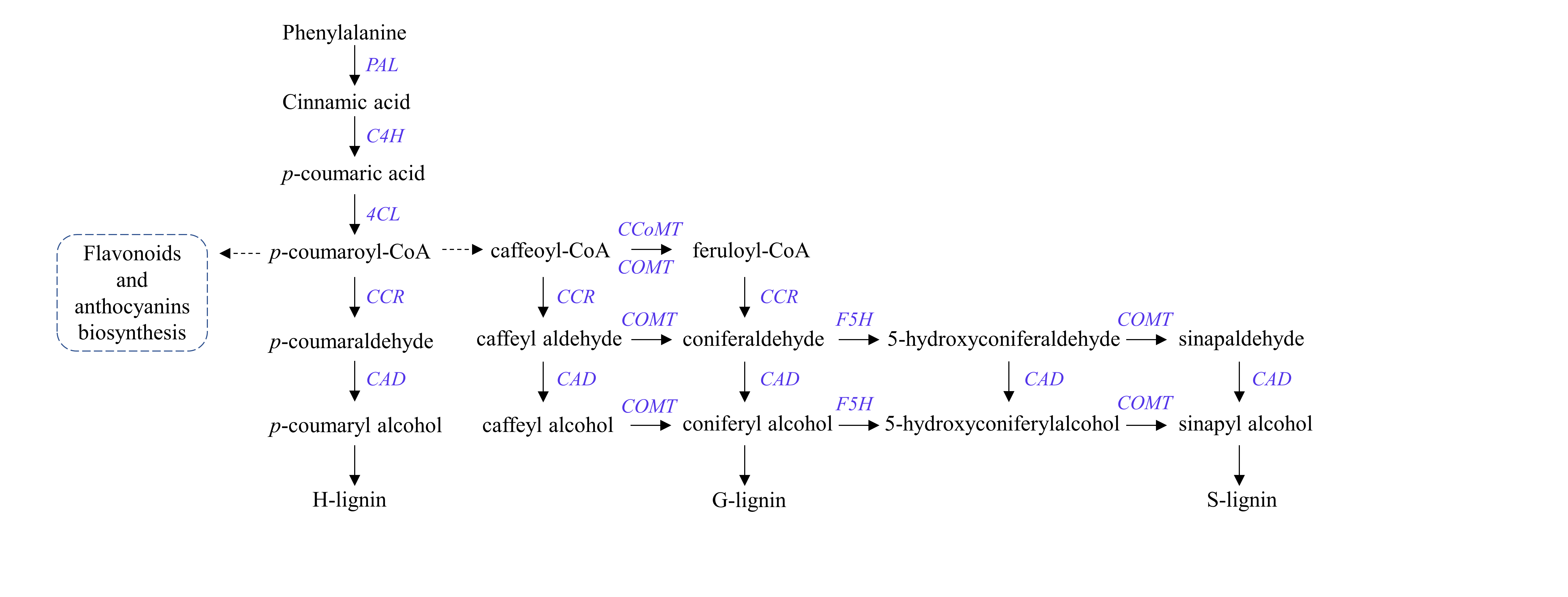


**Fig S5.** Lignin biosynthesis pathway.

**Fig S6**

**
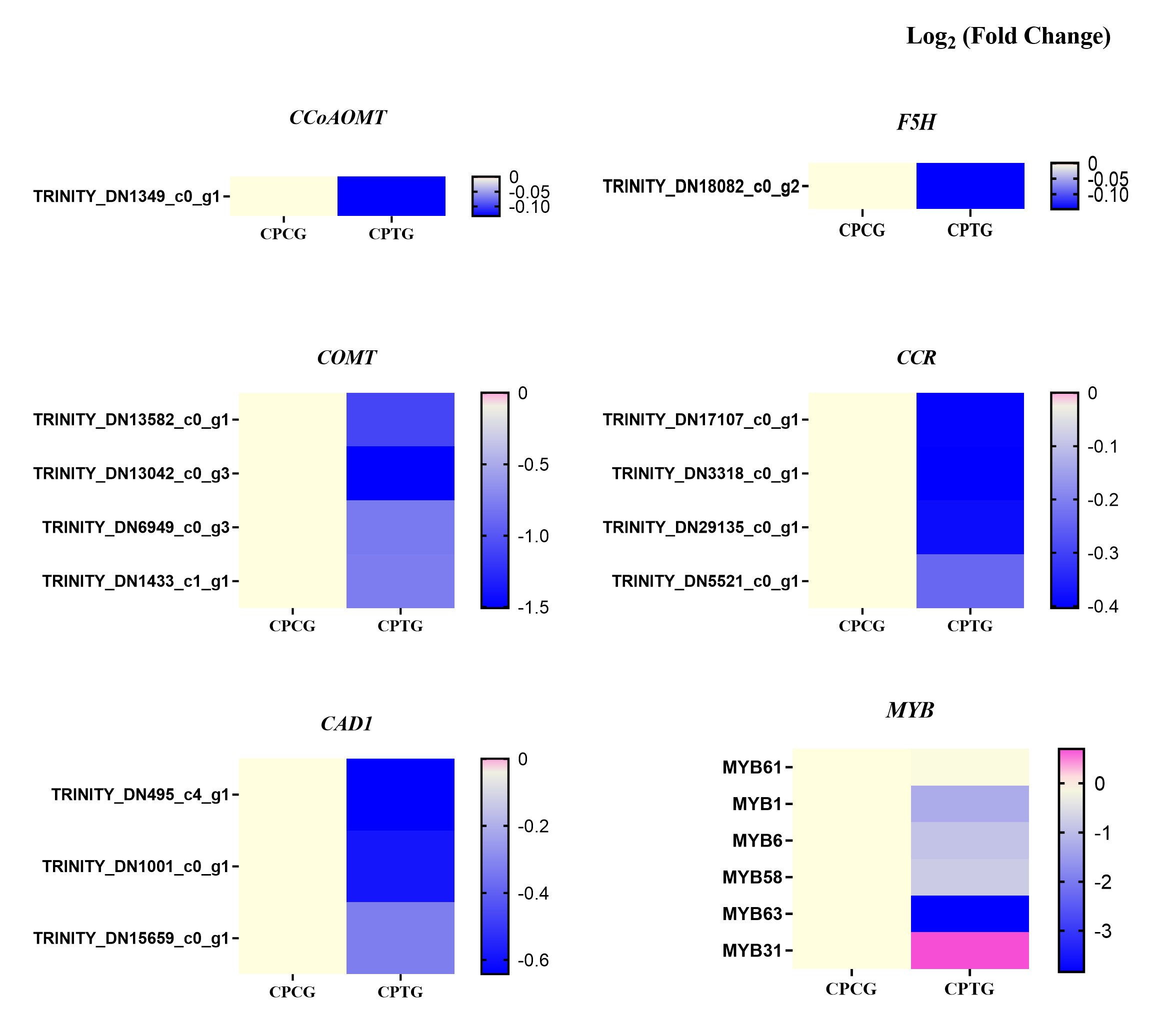
**

**Fig S6.** Expression level changes of DEGs and DETFs related to lignin biosynthesis pathway.

**Table S1** The standard curves for the detection of polyphenols

| **Polyphenol standards** | **RT/min** | **Linear regression equation** | **Linear range (μg/mL)** | **Correlation coefficient** |
| --- | --- | --- | --- | --- |
| Cyanidin-3-*O*-galactoside | 11.82 | *y*=19.572*x*+12.370 | 5~200 | 0.9998 |
| Procyanidin B1 | 14.13 | *y*=18.970*x*-48.056 | 10~400 | 0.9994 |
| (+) - Catechin | 17.05 | *y*=18.525*x*-15.946 | 20~400 | 0.9996 |

**Table S2** Single-factor experimental design for the five elicitors.

| **Elicitors** | **Concentration linear range** | **Addition time** |
| --- | --- | --- |
| 5-ALA | 0~100 μM | 1^st^, 2^nd^, 4^th^, 6^th^ |
| MeJA | 0~400 μM | 2^nd^, 4^th^, 6^th^ |
| SA | 0~100 μM | 2^nd^, 4^th^, 6^th^ |
| SNP | 0~40 μM | 2^nd^, 4^th^, 6^th^ |
| ROE | 0~200 μg/mL | 2^nd^, 4^th^, 6^th^ |

Note：all the experience groups were harvested when cultured on the 6^th^ day.

**Table S3** Primer sequences design.

| **Primer name** | **Primer sequence** |
| --- | --- |
| UBQ-F | GCCGACAACTCCTCCATC |
| UBQ-R | ATCTCCTCCGCTCATCATAAGA |
| TGA2-F | GCCACTTCCTCTCCTTCATTAC |
| TGA2-R | TCCACTATCCATAACCGACTCTC |
| LAR-F | GATTGTCGGAGCAACAGGTT |
| LAR-R | GGACGGACGAGAAGATAGGT |
| FLS-F | GCGTTGCCACTGATACTCTC |
| FLS-R | GATGCTCTGCTTGTGAACCTT |
| F3’5’H-F | ACCTACAACTACTCGGACATCA |
| F3’5’H-R | TTCTTAGCAAGGCACTCATCTC |
| ANR-F | ACCTATGAAGCAGAACGAAGATG |
| ANR-R | GTTGGCACCTGTTAGTTGAGA |
| 4CL-F | AAGACCATCCTAACCGTAGAAGAA |
| 4CL-R | CTTATGACCGTGCTGCTTGAT |
| NPR1-F | GGGCGTACAGGTTTAGTTGAG |
| NPR1-R | AGGTGGAGAGCATCCTATAATACT |
| PR1-F | ACGCCCAGAGGTTGTTGA |
| PR1-R | TGCCGCCACTACACTCAG |
| TGA6-F | GCTGCTCATTCCAACTCCTT |
| TGA6-R | TCTCACAGTCTCCACACCAA |

**Table S4** The comprehensive experiment results and analysis of different concentration combinations on total polyphenol content in *C. paliurus* cells.

| **No.** | **Treatment** | | | **Biomass (g/L)** | **Total polyphenol content (mg/g)** | **Total polyphenol yield (mg/L)** |
| --- | --- | --- | --- | --- | --- | --- |
|  | **A (SA)** | **B(5-ALA)** |  | |  |  |
| 1 | A_1_ | B_1_ | 15.550±0.42^aA^ | | 7.045±0.26 ^abAB^ | 109.494±1.08^abcABC^ |
| 2 | A_1_ | B_2_ | 16.000±0.35^aA^ | | 6.490±0.01 ^bcAB^ | 103.843±2.52^bcdABC^ |
| 3 | A_1_ | B_3_ | 16.250±0.00^aA^ | | 5.485±0.21^cdBC^ | 89.131±3.56^cdeBCD^ |
| 4 | A_2_ | B_1_ | 16.125±0.17^aA^ | | 7.090±0. 6^abAB^ | 114.380±11.06^abAB^ |
| 5 | A_2_ | B_2_ | 16.250±0.35^aA^ | | 7.945±0.6 ^bAB^ | 129.213±12.58^aA^ |
| 6 | A_2_ | B_3_ | 16.125±0.53^aA^ | | 6.395±0.19^bAB^ | 103.170±6.47^bcdABC^ |
| 7 | A_3_ | B_1_ | 15.705±0.37^aA^ | | 5.355±0.11 ^cdBC^ | 84.080±0.34^deCD^ |
| 8 | A_3_ | B_2_ | 16.125±0.17^aA^ | | 6.210±0.96^bcAB^ | 100.221±16.60^bcdABCD^ |
| 9 | A_3_ | B_3_ | 16.105±0.36^aA^ | | 4.4250±0.14^cD^ | 71.291±3.99^eD^ |

Note：A1, A2, A3: 25 μM, 50 μM, 100 μM of SA; B1, B2, B3: 25 μM, 50 μM, 100 μM of 5-ALA.

**Table S5** Analysis of variance on test factors.

| **Source of variation** | **Sum of squares of deviations** | **Freedom** | **Mean square** | ***F value*** | ***P value*** |
| --- | --- | --- | --- | --- | --- |
| Intercept | 715.428 | 1 | 715.428 | 2947.789 |  |
| A (SA) | 1.994 | 2 | 5.497 | 22.649 | ＜0.01**^**^** |
| B (5-ALA) | 5.773 | 2 | 2.887 | 11.893 | ＜0.05**^*^** |
| Interaction A×B | 1.549 | 4 | 0.387 | 1.596 |  |
| Error | 2.184 | 9 | 0.243 |  |  |
| Total variation | 735.929 | 18 |  |  |  |

Note：F_0.05_ (2,9)=4.26，F_0.01_ (2,9)=8.02. “**” indicates the difference is very significant, P＜0.01; “*” indicates the difference is significant, *P*＜0.05.

**Table S6** The analysis of multiple comparison.

| **Factor** | | **A** | | | **B** | | |
| --- | --- | --- | --- | --- | --- | --- | --- |
| Treatment | | A2 | A1 | A3 | B2 | B1 | B3 |
| Significance | 0.05 | a | b | b | a | b | c |
|  | 0.01 | A | b | B | A | B | B |

Note：A1, A2, A3: 25 μM, 50 μM, 100 μM of SA；B1, B2, B3: 25 μM, 50 μM, 100 μM of 5-ALA.

**Table S7** Summary sheet of the identified differential polyphenols and its KO_ID in KEGG database

| **Composition** | **KO_ID** | **Fold change of CPTG/CPCG** | **Trends** |
| --- | --- | --- | --- |
| Dihydrokaempferol | Ko00941 | 13.15* | up |
| Eriodictyol | Ko00941 | 0.27 | down |
| Hesperetin | Ko00941 | 6.88* | up |
| Naringenin | Ko00941 | 0.37 | down |
| Phloretin | Ko00941 | 5.16* | up |
| Prunin | Ko00941 | 2.47* | up |
| Quercetin | Ko00941; Ko00944 | 2.36* | up |
| Benzoic acid | Ko00360 | 9.07* | up |
| Salicylic acid | Ko00360; Ko04075 | 1.03 | up |
| L-Phenylalanine | Ko00360; Ko00940 | 1.65 | up |
| Pyruvic acid | Ko00360 | 1.61 | up |
| P-coumaric acid | Ko00940 | 1.98 | up |
| Caffeic acid | Ko00940 | 10.60* | up |
| Hyperoside | Ko00941 | 2.09 | up |
| Cyanidin-3-*O*-galactoside | Ko00940 | 22.06* | up |
| Catechin | Ko00941; Ko00360 | 15.45* | up |
| Quercetin-3β-D-glucoside | Ko00940 | 2.21 | up |
| Kaempferol 3-glucuronide | Ko00940; Ko00941 | 1.59 | up |
| Vitexin 2"-*O*-p-coumarate | Ko00360 | 25.10* | up |
| Taxifolin 7-galactoside | Ko00940 | 0.26 | down |
| Maritimetin | Ko00941 | 0.20 | down |

Note: “*” indicates that the difference multiple is significant.

**Table S8** The assembly statistic results of transcript and unigenes.

| **Item** | **Transcript** | **Unigene** |
| --- | --- | --- |
| Total Length (bp) | 234653324 | 72795281 |
| Sequence Number | 176857 | 71141 |
| Max. Length (bp) | 27518 | 27518 |
| Mean Length (bp) | 1326.80 | 1023.25 |
| N50 (bp) | 1995 | 1595 |
| N50 Sequence No. | 37267 | 12969 |
| N90 (bp) | 569 | 425 |
| N90 Sequence No. | 120108 | 50688 |
| GC % | 41.22 | 40.20 |

Note：N50: All sequences are arranged from long to short, and the sequence length is added in this order. When the added length reaches 50% of the total length of the sequence, the length of the last sequence is called N50; N90 (bp): All sequences are arranged from long to short, and the sequence length is added in this order. When the added length reaches 90% of the total length of the sequence, the length of the last sequence is called N90.
